# The Composite Material of (PEDOT-Polystyrene Sulfonate)/Chitosan-AuNPS-Glutaraldehyde/as the Base to a Sensor with Laccase for the Determination of Polyphenols

**DOI:** 10.3390/ma16145113

**Published:** 2023-07-20

**Authors:** Paweł Krzyczmonik, Marta Klisowska, Andrzej Leniart, Katarzyna Ranoszek-Soliwoda, Jakub Surmacki, Karolina Beton-Mysur, Beata Brożek-Płuska

**Affiliations:** 1 Department of Inorganic and Analytical Chemistry, Faculty of Chemistry, University of Lodz, Tamka 12, 91-403 Lodz, Poland; 2 Department of Materials Technology and Chemistry, Faculty of Chemistry, University of Lodz, Pomorska 163 Street, 90-236 Lodz, Poland; katarzyna.ranoszek.soliwoda@chemia.uni.lodz.pl; 3 Laboratory of Laser Molecular Spectroscopy, Faculty of Chemistry, Institute of Applied Radiation Chemistry, Lodz University of Technology, Wroblewskiego 15, 93-590 Lodz, Poland; jakub.surmacki@p.lodz.pl (J.S.);

**Keywords:** PEDOT, chitosan, Au nanoparticles, laccase, immobilization, electrochemical biosensor, differential pulse voltammetry, SEM, AFM, Raman

## Abstract

The described research aimed to develop the properties of the conductive composite /poly(3,4-ethylenedioxy-thiophene-poly(4-lithium styrenesulfonic acid)/chitosan-AuNPs-glutaraldehyde/ (/PEDOT-PSSLi/chit-AuNPs-GA/) and to develop an electrochemical enzyme sensor based on this composite material and glassy carbon electrodes (GCEs). The composite was created via electrochemical production of an /EDOT-PSSLi/ layer on a glassy carbon electrode (GCE). This layer was covered with a glutaraldehyde cross-linked chitosan and doped with AuNPs. The influence of AuNPs on the increase in the electrical conductivity of the chitosan layers and on facilitating the oxidation of polyphenols in these layers was demonstrated. The enzymatic sensor was obtained via immobilization of the laccase on the surface of the composite, with glutaraldehyde as the linker. The investigation of the surface morphology of the GCE/PEDOT-PSSLi/chit-AuNPs-GA/Laccase sensor was carried out using SEM and AFM microscopy. Using EDS and Raman spectroscopy, AuNPs were detected in the chitosan layer and in the laccase on the surface of the sensor. Polyphenols were determined using differential pulse voltammetry. The biosensor exhibited catalytic activity toward the oxidation of polyphenols. It has been shown that laccase is regenerated through direct electron transfer between the sensor and the enzyme. The results of the DPV tests showed that the developed sensor can be used for the determination of polyphenols. The peak current was linearly proportional to the concentrations of catechol in the range of 2–90 μM, with a limit of detection (LOD) of 1.7 μM; to those of caffeic acid in the range of 2–90 μM, LOD = 1.9 μM; and to those of gallic acid in the range 2–18 μM, LOD = 1.7 μM. Finally, the research conducted in order to determine gallic acid in a natural sample, for which white wine was used, was described.

## 1. Introduction

Sensors and biosensors are among the most interesting tools of modern analytical chemistry. The essence of the proper operation of sensors is to design them for the needs of specific analyses. This requires the use of a variety of materials and, sometimes, the development of new materials to play a specific role in the operation of the sensor as a whole. In the described research, a new type of composite, which combines the conductive PEDOT-PSSLi layer with a chitosan layer, was developed.

Chitosan is a substance of natural origin. It is a polysaccharide that is obtained through the partial deacetylation of chitin. It is characterized by a very high level of biocompatibility. Chitosan is a substance used in medicines and may safely come into direct contact with the body, e.g., some materials made with chitosan are used to stop bleeding and as burn dressings [1,2]. This feature is very desirable in systems that are designed to work with natural samples because they increase the durability of the sensor and, at the same time, have no negative impact on the tested sample. Moreover, chitosan is characterized by its excellent film-forming ability and good mechanical strength. Although chitosan is a non-conductive biomaterial, it can be used together with conductive polymers, such as polyaniline [3,4], polypyrrole [5,6], and PEDOT [7,8,9], and polymer/chitosan coatings can be used in the construction of sensors with electrochemical detection. Very often, in the developed sensors, solid electrodes such as Au, Pt, or various types of carbon are used, and they are modified with layers of conductive polymers.

In many applications, the properties of PEDOT turned out to be better than those of polypyrrole and polythiophene. Krosa et al. [10] proved in their studies that polypyrrole can only be used as a biosensor component for a very short time, while PEDOT, in the same applications, turned out to be a component that was suitable for continuous use. According to the authors, this was due to the fact that PEDOT has greater electrochemical stability than polypyrrole. In the case of systems containing biochemical objects, it is very important that PEDOT has low toxicity in comparison to, for example, polyaniline, whose degradation products are carcinogenic [11]. PEDOT doped with polystyrene sulfonic acid salts is also very widely used. It is a material in which the anions doping the conductive polymer are permanently bound to the PEDOT layer, which increases the durability of the entire system and does not allow for a complete reduction of the PEDOT. In the described tests, glassy carbon electrodes (GCEs), modified with the PEDOT-PSSLi layer, were used. This type of material was previously used to modify platinum and GCE electrodes [12,13] and as a basis for the construction of enzymatic sensors [14,15].

In order to change its electrical properties, the chitosan was doped with AuNPs, which allowed us to obtain conductivity in the entire layer. In addition, AuNPs were used for the direct oxidation of laccase on the sensor surface. Gold nanoparticles are materials characterized by very good electrical conductivity and a very high degree of biocompatibility; AuNP suspensions are used in various medical therapies. The sizes of gold nanoparticles depend on the method of their synthesis. In the described research, nanoparticles synthesized via reaction with sodium borohydride were used [16]. As a result of this reaction, nanoparticle suspensions with sizes of 3–5 nm were obtained. The disadvantage of chitosan layers is their susceptibility to dissolution in aqueous solutions, which reduces the durability of sensors based on this material. In order to eliminate this drawback, the chitosan layer was modified through cross-linking with glutaraldehyde, which made the layer resistant to dissolution.

It is known from the literature reports that chitosan is very suitable for immobilizing biomolecules on its surface [11,12,13,14,15,16,17,18,19]. Chitosan molecules contain amino groups and carboxyl groups in their structure. As a result, it is possible to immobilize various biological objects, such as enzymes, to one or the other functional group by forming a covalent bond. It is one of the most frequently used polymers of natural origin in biosensors [17,20,21]. In the described research, the authors decided to immobilize laccase by linking the amino groups of the enzyme and the amino groups of chitosan, using glutaraldehyde as a linker. The use of glutaraldehyde enabled the formation of covalent bonds between laccase and chitosan. Compared to traditional chromatographic methods, electroanalytical techniques based on oxidation–reduction reactions have many advantages, such as simplicity, low cost, high stability and sensitivity, fast response, and excellent repeatability [22,23]. If electrochemical sensors are used for electroanalytical methods, it is possible to obtain analytical techniques for which preliminary sample preparation will not be necessary. This allows us to design systems that will be promising analytical tools for the analysis of real samples.

The purpose of the research was to develop a conductive electrode material based on chitosan, which could be the basis for the production of an electrochemical sensor with an immobilized enzyme. The enzyme chosen to be immobilized was laccase, which was bound to the substrate through the formation of a covalent bond. This method of immobilization is the most effective because it eliminates the problem of leaching the enzyme from the layer. Glutaraldehyde was used to cross-link the structure of the chitosan layer, which increased its durability, and AuNPs were used to increase the electrical conductivity of the chitosan layer. In the first stage of the research, the focus was on obtaining a composite material that would ensure the best electrochemical properties and the possibility of attaching an enzyme molecule. In the second stage of the research, a method for laccase immobilization on the surface of the composite material, using glutaraldehyde, was developed. The electrochemical properties were characterized with cyclic voltammetry in ferricyanides solutions. The electrocatalytic oxidation of polyphenols was tested using a sensor in catechol solutions. The obtained materials were characterized using SEM, EDS, Raman spectroscopy, and cyclic voltammetry. The last stage involved tests for the determination of selected polyphenols in aqueous solutions, in order to investigate the possibility of their use in electroanalysis. The possibility of the determination of polyphenols in a natural sample, for which white wine was used, was also presented.

## 2. Materials and Methods

All of the chemical reagents were analytically pure and were used without further purification. Laccase, 3,4-ethylenedioxy-thiophene, catechol, caffeic acid, gallic acid, and Rhodanine were supplied by Sigma-Aldrich (St. Louis, MO, USA). Chitosan was obtained from Across Organic (Geel, Belgium) and HAuCl_4_·3H_2_O was obtained from AlfaAesar (Kandel, Germany). The 25% solution of glutaraldehyde, potassium ferrocyanide, sodium chloride, citric acid, trisodium citrate, potassium chloride, acetic acid, disodium hydrogen orthophosphate dodecahydrate (Na_2_HPO_4_·12H_2_O), potassium dihydrogen orthophosphate, and sodium hydroxide were supplied by POCH Gliwice. All of the solutions were prepared just before use, with water purified using the Millipore (Milli-Q) system. Laccase was stored at 4 °C.

**Instrumentation**

The measuring equipment comprised a PAR 273A potentiostat (EG&G Princeton Applied Research Company, Princeton, NJ, USA) and a computer with CorrWare 2.9 and CorrView 2.9 software (Scribner Associates, Inc. Southern Pines, NC, USA). All of the electrochemical measurements were carried out in a three-electrode cell. A modified glassy carbon electrode was used as a working electrode, a saturated calomel electrode (SCE) was used as a reference electrode, and a platinum mesh was used as a counter electrode. The morphology of sensors was investigated using an Atomic Force Microscope (DIMENSION ICON ScanAsyst, BRUKER, Billerica, MA, USA). The measurements were taken using the scanning probe TESPA-New 09 in tapping mode. The surface morphology was also investigated with electron microscopy using a High-Resolution Scanning Electron Microscope (HR-SEM, FEI Nova NanoSEM 450, Hillsboro, OR, USA) equipped with a CBS detector for the detection of backscattered electrons. The chemical composition analysis was performed using the energy-dispersive spectrometer (EDS, EDAX/AMETEK, Materials Analysis Division, Model Octane Super, Mahwah, NJ, USA). A WITec alpha 300 RSA+ confocal microscope was used to record Raman spectra. The configuration of the experimental setup was as follows: the diameter of fiber, 50 μm for 532 nm, a monochromator Acton-SP-2300i, and a CCD camera Andor Newton DU970-UVB-353 for 532 nm. The excitation line was focused on the sample through a 40× dry objective (Nikon, objective type CFI Plan Fluor C ELWD DIC-M, numerical aperture (NA) of 0.60 and a 3.6–2.8 mm working distance). The laser excitation power was 10 mW at 532 nm for pure components (laccase and chitosan), and 2.7 mW for samples deposited on the electrodes, with an integration time of 0.5 s and 10 accumulations. Data acquisition and processing were performed using WITec Project Plus software ver. 4.1. The cosmic rays were removed from each Raman spectrum (model: filter size, 2; dynamic factor, 10), and for the smoothing procedure, the Savitzky–Golay method was also implemented (model: order, 4; derivative, 0). The baseline corrections of Raman spectra were performed using WITec Project Plus and OriginPro. 

**Synthesis of gold nanoparticles (AuNPs)**

The synthesis of gold nanoparticles (AuNPs) was carried out by mixing 30 mL of distilled water, 1.8 mL of 0.0025 M Au (III), and 1.5 mg of sodium borohydride NaBH_4_ [16]. During preparation and reaction, the solution was continuously stirred on a magnetic stirrer at room temperature. The reaction product was a violet–blue suspension of gold nanoparticles. The SEM image of the obtained gold nanoparticles and the histogram describing the size of the nanoparticles are shown in Figure S1 of the Supplementary Materials. 

**Preparation of composite layers for GCE/PEDOT-PSSLi/chitosan-AuNPs-GA/**

The following procedure was used for the modification of the glassy carbon electrode. The first stage involved cleaning the working electrode surface. The glassy carbon electrode was carefully polished with an aqueous alumina slurry (0.5 μm) on a microcloth pad, and then thoroughly washed with double-distilled water.

(a) The layer of GCE/PEDOT-PSSLi was obtained by means of potentiostatic electrolysis at potential E = 1 V and time t = 20 s. The polymerization solution contained 0.002 mol/dm^3^ EDOT and 0.1 mol/dm^3^ PSSLi. This procedure was described in previous publications [12,14].
(b) For the layer of GCE/PEDOT-PSSLi/chitosan-AuNPs-GA, a solution containing 0.5 mL of AuNP suspension, 0.25 mL of 1% chitosan solution (in 0.05 M acetic acid), and 5 µL of 2.5% glutaraldehyde solution was prepared. The solution was stirred on a magnetic stirrer, and then 7 µL of the obtained solution was taken and spotted on the surface of the GCE/PEDOT-PSSLi electrode. The electrode was left for 1 h at the room temperature.

**Preparation of the GCE/PEDOT-PSSLi/chitosan-AuNPs-GA/laccase sensor**

A solution containing 12 mg laccase and 30 µL of 2.5% glutaraldehyde solution in 1 mL phosphate–citrate buffer (pH = 5.0) was prepared. The GCE/PEDOT-PSSLi (chitosan-AuNPs-GA) electrode was immersed in this solution and left for 3 h at 4 °C. Then, the electrode was rinsed with PBS solution and distilled water in order to remove the unbound enzyme.

Due to the durability of the coating, the sensor must not be allowed to dry completely; therefore, the prepared electrode was stored at 4 °C and immersed in a small amount of phosphate–citrate buffer (pH = 5.0). This method of storing the sensors ensured their stability for 30 days, with a signal drop of no more than 10%.

## 3. Results

**Figure 1:**
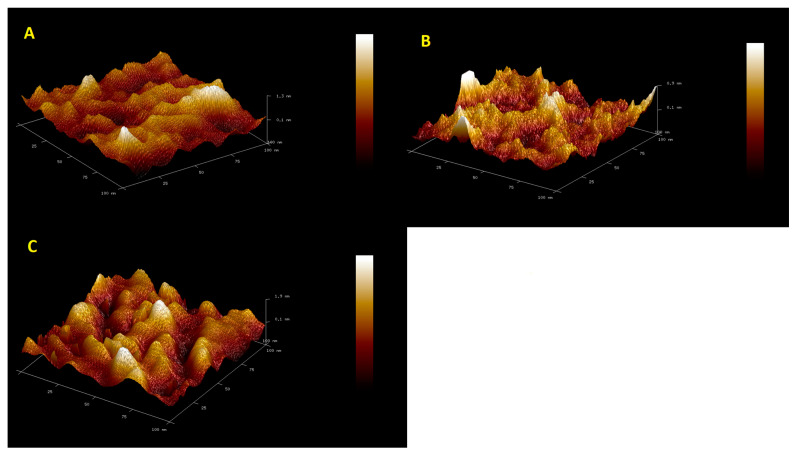
AFM microscopy images for (**A**) GCE/PEDOT-PSSLi/Chit-GA, (**B**) GCE/PEDOT-PSSLi/Chit-AuNPs-GA, and (**C**) GCE/PEDOT-PSSLi/Chit-AuNPs-GA/Laccase.

Figure 1 shows AFM microscopy images for the GCE/Chit-GA, GCE/Chit-AuNPs-GA, and GCE/Chit-AuNPs-GA/laccase layers. Table 1 shows the roughness parameter values calculated for these surfaces. The differences between the layers of chitosan and those of chitosan doped with AuNPs are slight. The effect of doping with gold nanoparticles on the morphology of the chitosan surface was small: their addition caused a slight smoothing of the chitosan’s surface. Small differences in the morphology of both layers may result from the fact that the AuNP doping takes place in the entire volume of the chitosan layer and not only on the surface of this layer. The next image shows clear differences in the surface structure, caused by the immobilization of the enzyme. This is confirmed by a clear increase in the surface roughness value (Table 1). This image also shows that the surface of the sensor is evenly coated with the enzyme.

**Figure 2:**
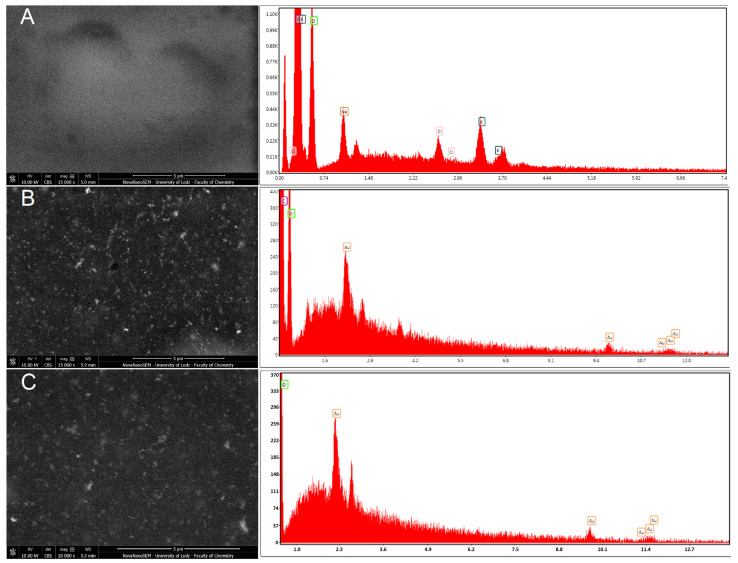
SEM images with EDS spectra of chitosan layers containing AuNPs: (**A**) GCE/PEDOT-PSSLi/Chit-GA, (**B**) GCE/PEDOT-PSSLi/Chit-AuNPs-GA, and (**C**) GCE/PEDOT-PSSLi/Chit-AuNPs-GA/Laccase.

The surface morphology was also investigated using electron microscopy SEM with a CBS detector for the detection of backscattered electrons. The use of the CBS detector allowed us to obtain the “Z-contrast image”, which is directly related to the atomic numbers of the elements that are the components of the material. Hence, the CBS detector was very useful for both the surface morphology investigation and the confirmation of the presence of metallic nanoparticles as a dispersed phase in the material matrix. The CBS detects more signals from atoms with higher atomic numbers (AuNPs), and these elements can be seen as brighter spots/areas in the resulting image (Figure 2B,C) compared with the matrix material (Figure 2A), which has a darker color in the image. An analysis of the HR-SEM images revealed the presence of AuNPs homogenously distributed within the material (Figure 2B,C). The EDS analysis confirmed the presence of AuNPs in the sensors as peaks characteristic of Au, at Lα = 9.712 eV and M = 2.120 eV (Figure 2B,C). The small EDS signals from Au were caused by the small size of single AuNPs, and the fact that they are embedded in the matrix. However, the EDS composition analysis and the CBS morphology images confirmed the presence and homogenous distribution of AuNPs in the sensors.

**Figure 3:**
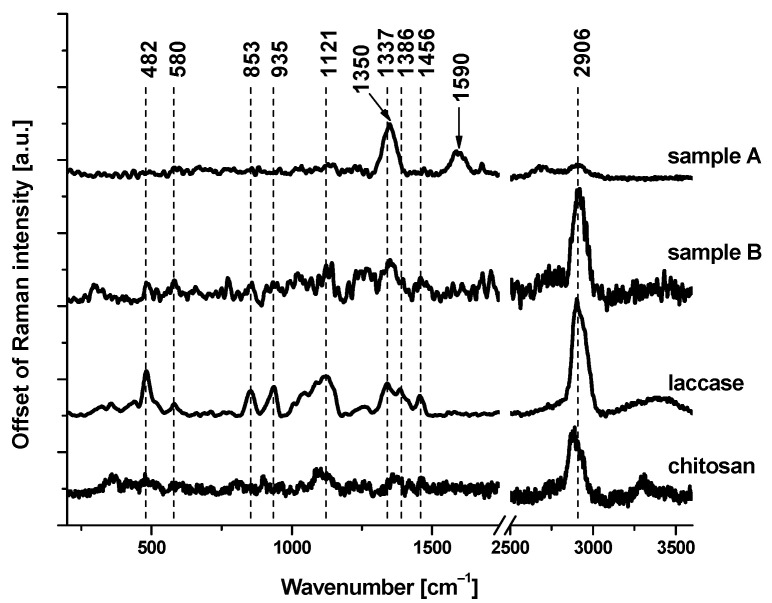
Raman spectra of chitosan, laccase, and two electrodes: without (sample A) and with (sample B) laccase.

In order to confirm the presence of laccase on the electrode, we performed a Raman spectroscopy analysis. Raman spectroscopy is a non-destructive analytical technique in which inelastically scattered light is used to obtain information about the vibrational energy modes of the analyzed samples. Figure 3 shows the Raman spectra of reference chemical compounds (laccase and chitosan) and chemicals deposited on the electrodes: without (sample A) and with (sample B) laccase. Characteristic Raman bands, at 482, 580, 853, 935, 1121, 1350, 1337, 1386, 1456, and 2906 cm^−1^, correspond to laccase. A detailed inspection of Figure 3 demonstrates that the most significant Raman bands attributed to laccase are present in the Raman spectrum of the electrode with that enzyme. Broad Raman bands, observed in sample A at 1350 and 1590 cm^−1^, correspond to amorphous carbon sp^2^ (D-band) and amorphous carbon sp^3^ (G-band) [24], respectively.

**Figure 4:**
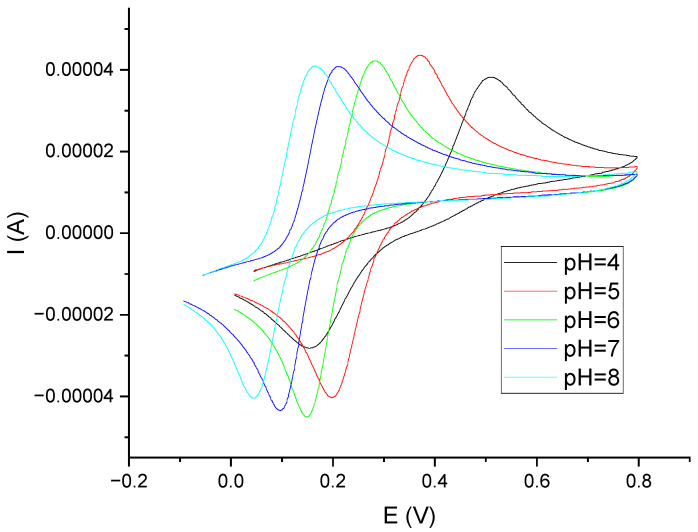
Voltammetric curves obtained on GCE in a solution of catechol C = 0.001 M in phosphate–citrate buffer for different pH values (v = 200 mV/s). pH = 4.0 (black), pH = 5.0 (red), pH = 6.0 (green), pH = 7.0 (blue), and pH = 8.0 (cyan).

**Figure 5:**
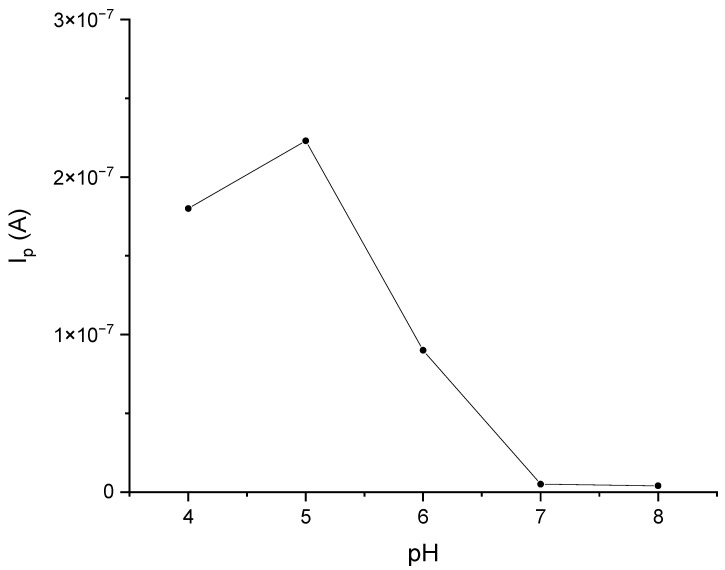
The effect of pH on the response of the GCE/PEDOT-PSSLi/Chit-AuNPs-GA/laccase sensor in catechol solutions 0.001 M in phosphate–citrate buffer. Dependences of the current peak on pH.

Laccase is the enzyme that shows the highest activity at pH = 5 [25,26]. Polyphenols, on the other hand, are compounds whose electrochemical oxidation reactions depend on the pH of the environment. Voltammetric curves of catechol in phosphate–citrate buffer solutions at pH values of 4, 5, 6, 7, and 8 are shown in Figure 4. Analogous curves for gallic acid and caffeic acid are provided in the Supplementary Materials (Figures S2 and S3). Based on these data, a phosphate–citrate buffer environment with pH = 5 was adopted for further research. In order to confirm the validity of this assumption, DPV measurements were performed, using a laccase sensor for solutions of catechol in phosphate–citrate buffers at pH values of 4, 5, 6, 7, and 8. Figure 5 shows the dependence of the peak current on pH. As can be seen, the highest peak currents were observed in the solutions when pH = 5.

In addition to the pH value, other parameters of the sensor manufacturing process were also optimized. The thickness of the produced chitosan layer was optimized by determining the volume of the chitosan solution applied to the electrode. Too-thick layers peeled off after drying and fell off of the substrate. Moreover, thick layers of non-conductive chitosan deteriorated the electrochemical properties of the electrode. The application of chitosan solution drops in different volumes on the surface of the electrode was tested. Droplets of 4, 5, 6, 7 and 8 μL were applied. The layers prepared by applying 7 µL of chitosan solution to the electrode (electrode diameter 3 mm) produced the best results and were used for further studies. Droplets of less than 7 µL did not cover the entire electrode. On the other hand, droplets of more than 7 µL formed thicker layers of lower conductivity, with a tendency to exfoliate.

In the next stage, the influence of gold nanoparticles on the electrochemical properties of the chitosan layers was investigated. The amount of the AuNP mixture added to the chitosan solution was optimized. The addition of AuNPs was intended to increase the electrical conductivity of the layer and improve its electrochemical properties. Too much AuNP suspension added to the chitosan solution diluted it, and, as a result, a too-thin chitosan layer formed. On the other hand, the layer obtained from a solution with a smaller amount of AuNPs demonstrated worse electrical conductivity. The assumed optimal composition contained 0.5 mL of AuNP suspension, 0.25 mL of 1% chitosan solution, and 5 µL of 2.5% glutaraldehyde solution. The effect caused by the addition of nanoparticles was assessed using the measurements from cyclic voltammetry of the tested electrodes in solutions of ferricyanides. For this purpose, voltammetric measurements were performed for the following three sensors: GCE/PEDOT-PSSLi, GCE/PEDOT-PSSLi/Chit-GA, and GCE/PEDOT-PSSLi/Chit-AuNPs-GA.

**Figure 6:**
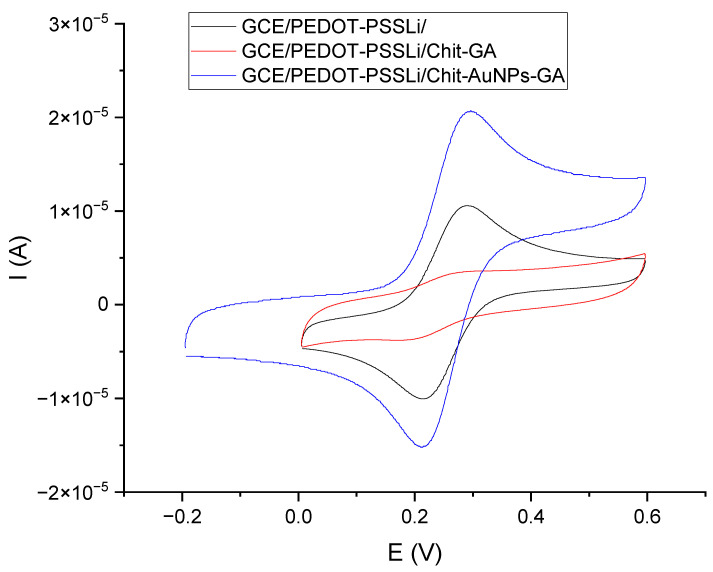
Voltammetric curves for sensors obtained in solutions of 0.002 M K_2_Fe[(CN)_6_]_4_ in 2 M KCl (v = 200 mV/s). GCE/PEDOT-PSSLi/Chit-AuNPs-GA (black), GCE/PEDOT-PSSLi/Chit-GA (red), and GCE/Chit-AuNPs-GA (blue).

All measurements were carried out in solutions of ferrocyanide, at a concentration of 0.002 M in 2 M KCl (Figure 6). Both peak currents and differences in peak potential were evaluated. In the case of the first, the GCE/PEDOT-PSSLi sensor, the peak potential difference was 76 mV, and the peaks were symmetrical and well-formed. In the case of the GCE/PEDOT-PSSLi/Chit-GA sensor, the chitosan layer without the addition of nanoparticles worsened the properties of the electrode. The currents were very low, and the difference in peak potential was 119 mV. This is understandable because the PEDOT-PSSLi layer provides better conductivity and facilitates redox processes, while chitosan cross-linked via glutaraldehyde is an insulator and will deteriorate the properties of the electrode.

For the GCE/PEDOT-PSSLi/Chit-AuNPs-GA sensor, the highest current values were obtained, the difference in peak potential was 83 mV, and the peaks were symmetrical and well-shaped. This proves that the addition of gold nanoparticles increased the conductivity of the chitosan layer.

Thus, it can be concluded that the doping of the chitosan layer with gold nanoparticles significantly increased the conductivity of the layer and, at the same time, the presence of AuNPs allowed for a rapid charge exchange through the interface of the solution/chitosan doped with AuNPs.

The volume of glutaraldehyde solution added to the mixture of AuNPS and chitosan was optimized. Glutaraldehyde cross-links the chitosan layer by binding amino groups, but using too much aldehyde will block all amino groups, which will prevent the immobilization of the enzyme at the next stage of work. The level of saturation of the chitosan layer with glutaraldehyde was assessed using the immobilization of laccase on the prepared layers. The research began with the addition of 240 µL of 2.5% solution of glutaraldehyde to the mixture of AuNPs with chitosan. This amount completely saturated all amino groups in chitosan and prevented enzyme immobilization. In the next tests, the amount of glutaraldehyde was reduced successively to 120, 60, and 30 µL, until a layer with immobilized laccase was obtained. For the volumes of 60 and 30 µL, the oxidation current of polyphenols was obtained. This means that, after using such volumes of glutaraldehyde, free amino groups, which are capable of binding to the enzyme, remained in the chitosan structure. We decided to use 30 µL of 2.5% glutaraldehyde solution for the cross-linking of chitosan layers.

In the last stage of sensor development, the amount of immobilized enzyme was optimized. The results were evaluated on the basis of measurements of cyclic voltammetry of the sensor in the catechol solution. The enzyme was immobilized from solutions containing 3.0, 6.0, 9.0, and 12 mg laccase/1 mL solution. The peak current grew with the increasing amount of immobilized enzyme, up to a layer obtained from a solution of 12 mg laccase/1 mL phosphate–citrate buffer (pH = 5.0). The layers obtained from solutions with a higher amount of laccase yielded a peak current of a similar value, but they dissolved more easily.

**Figure 7:**
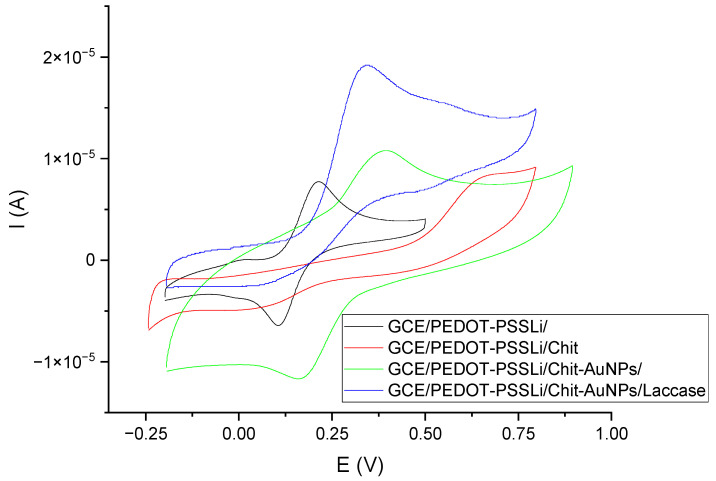
Voltammetric curves obtained in a solution of catechol C = 0.001 M (in phosphate–citrate buffer pH = 5.0) for sensors: GCE/PEDOT-PSSLi (black), GCE/PEDOT-PSSLi/Chit (red), GCE/PEDOT-PSSLi/Chit-AuNPs-GA (green), GCE/PEDOT-PSSLi/Chit-AuNPs-GA/laccase (blue).

Another characteristic is the comparison of the voltammetric curves of the developed sensor at the subsequent stages of its production. These studies were performed in catechol solutions (c = 0.001 M) in phosphate–citrate buffer at pH = 5. The results of these measurements are shown in Figure 7. Measurements were made on four sensors. Comparison showed the influence of individual sensor components on the catechol oxidation reaction. The first sensor was the GCE/PEDOT-PSSLi one, for which the oxidation peak potential is 0.214 V, and the peaks are symmetrical. The second sensor was the GCE/PEDOT-PSSLi/Chit one. In this case, the oxidation reaction was inhibited. The peak current was much lower, and the peak potential was shifted to about 0.680 V. This is due to the fact that the chitosan layer effectively insulates the electrode surface. The potential of the oxidation peak shifted to 0.675 V. The third sensor was the GCE/PEDOT-PSSLi/Chit-AuNPs one, which differs from the previous sensor only in the addition of AuNPs to the chitosan layer. As can be seen, this greatly improved the properties of the electrode. The oxidation peak potential, in this case, was 0.390 V. The fourth sensor was the complete GCE/PEDOT-PSSLi/Chit-AuNPs/Laccase system, for which the peak potential became 0.343 V, and the peak current had the highest value.

**Figure 8:**
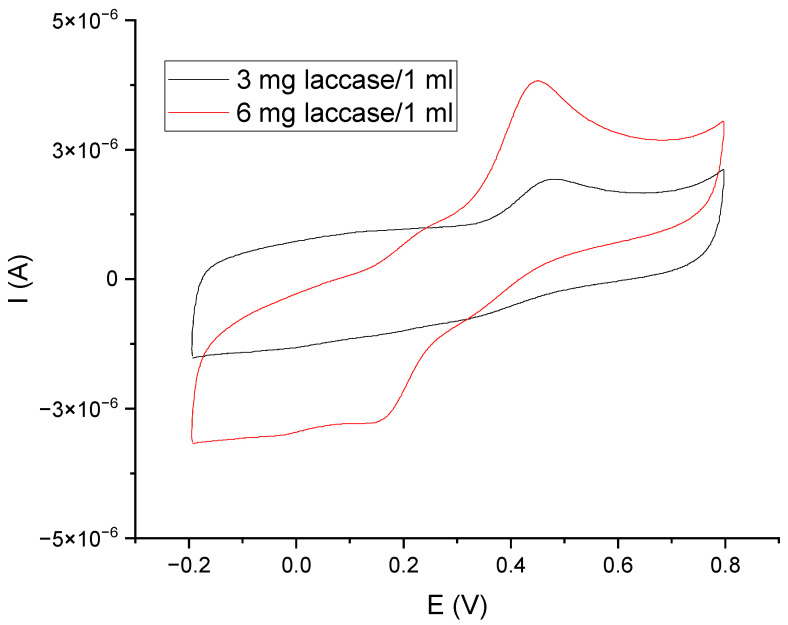
Voltammetric curves for GCE/PEDOT-PSSLi/Chit-AuNPs-GA sensors, obtained in a solution of catechol C = 9.09 E−05 M (in phosphate–citrate buffer pH = 5.0) and laccase in concentrations of 3 mg/1 mL (black) and 6 mg/1 mL (red). v = 200 mV/s.

The next step in the design of the sensor was to study the operation of the GCE/PEDOT-PSSLi/Chit-AuNPs-GA system in combination with laccase as a mediator in solution. Figure 8 shows the voltammetric curves made for the GCE/PEDOT-PSSLi/Chit-AuNPs-GA sensor in a catechol solution, with a concentration of C = 9.09 × 10^−5^ mol/dm^3^ in a phosphate–citrate buffer at pH = 5.0, with the addition of 3 mg laccase/1 mL (black curve) and 6 mg/1 mL (red curve). In this case, at a constant concentration of catechol, the value of the peak current depends on the concentration of laccase. This proves that laccase is involved in the oxidation of catechol as a mediator.

**Figure 9:**
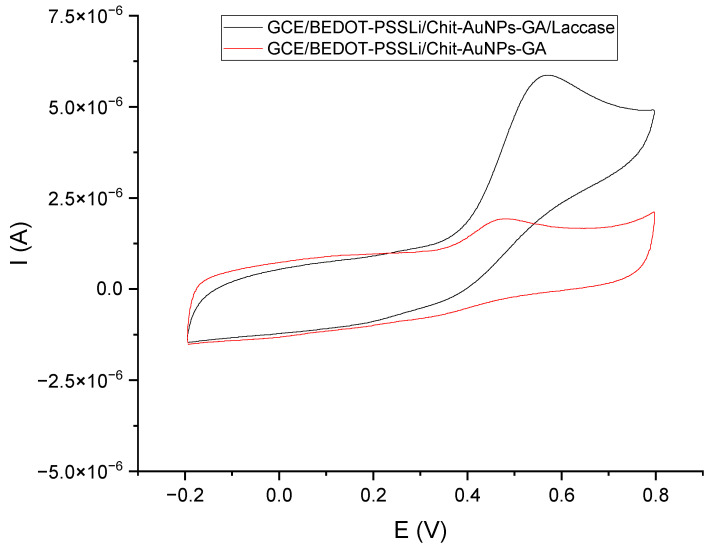
Voltammetric curves: black—GCE/PEDOT-PSSLi/Chit-AuNPs-GA/laccase sensor in a solution of catechol C = 9.09 E−05 M (in buffer pH = 5.0); red—GCE/PEDOT-PSSLi/Chit-AuNPs-GA sensor in a solution of catechol C = 9.09 E−05 M (in phosphate–citrate buffer pH = 5.0) and laccase in concentrations of 3 mg/1 mL.

The last step was to test the performance of the sensor with immobilized laccase (GCE/PEDOT-PSSLi/Chit-AuNPs-GA/laccase) with the GCE/PEDOT-PSSLi/Chit-AuNPs-GA sensor immersed in a solution containing laccase. Immobilization of the enzyme was carried out in a solution containing 12 mg laccase and 30 µL of 2.5% glutaraldehyde solution in 1 mL phosphate–citrate buffer (pH = 5.0). The voltammetric curve obtained in this measurement was compared with the curve obtained for the GCE/PEDOT-PSSLi/chit-AuNPs-GA sensor in a catechol solution with a concentration of C = 9.09 × 10^−5^ mol/dm^3^ (in phosphate–citrate buffer pH = 5.0), with the addition of 3 mg/1 mL (the red curve from Figure 9). As can be seen for the sensor with laccase immobilized on the surface, the obtained peak current was about six times higher than that without. Such a large catalytic effect of immobilized laccase makes the tested sensor a promising analytical tool for the determination of polyphenols.

Using the developed sensor (GCE/PEDOT-PSSLi/chit-AuNPs-GA/Laccase), catechol was determined using the amperometric method. The measurements were carried out in a phosphate–citrate buffer solution (pH = 5.0) at a potential of 0.6 V for 30 s while the solution was agitated. The amperometric curves of the measurements performed are shown in Figure S4 of the Supplementary Materials. The measurements were made five times, on a newly prepared sensor each time. The average values of currents from all five measurements were taken for the analysis. The measurements were performed for the concentration range from 1.96 × 10^−5^ to 9.09 × 10^−5^ mol/dm^3^. For the analysis of the results, the values of the currents read for the time t = 25 s were used. The R^2^ value was 0.9958, LOD = 9.5 µmol/dm^3^, and the linear range was 19–90 µmol/dm^3^.

**Figure 10:**
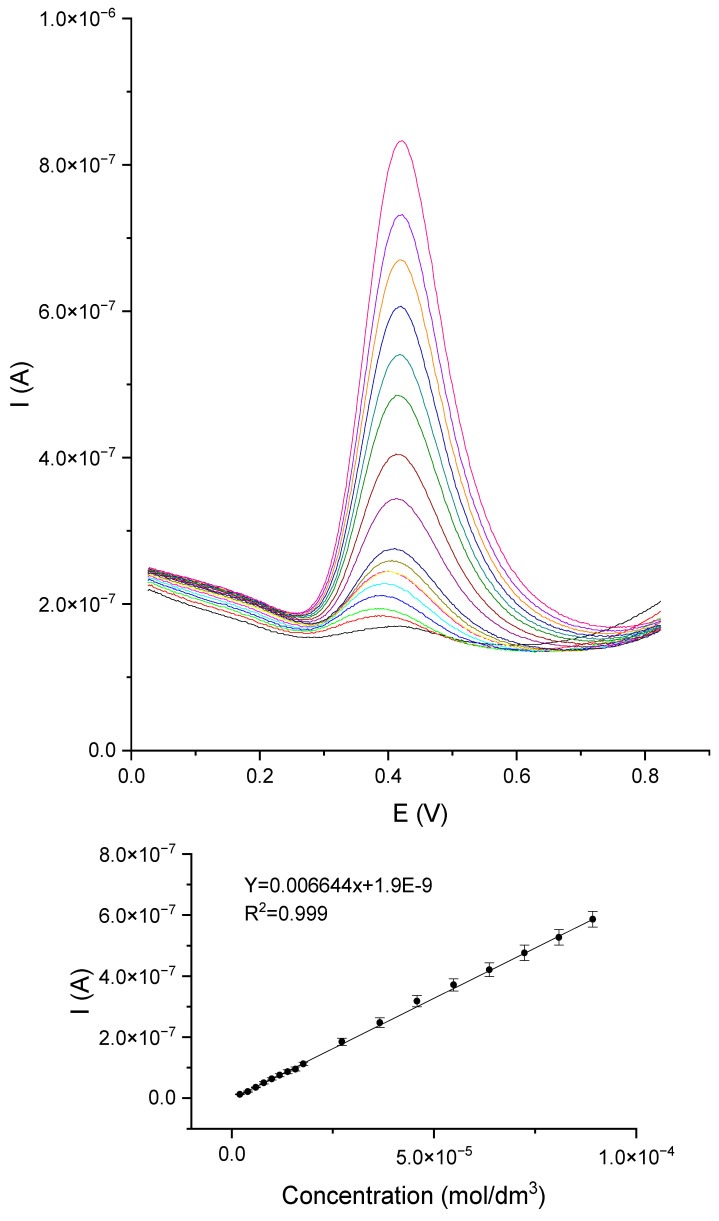
DPV voltammetry curves for the GCE/PEDOT-PSSLi/Chit-AuNPs-GA/laccase sensor in solutions of caffeic acid in phosphate–citrate buffer pH = 5.0.

The next step was to determine the polyphenols catechol, gallic acid, and caffeic acid using the DPV method. The measurements of DPV were carried out for the following parameters: potential range from 0.0 V to 0.8 V, potential jump 2 mV, duration of the jump 0.4 s, pulse amplitude 50 mV. The DPV voltammetry curves and the caffeic acid standard line are shown in Figure 10. The other polyphenol curves and standard lines are provided in the Supplementary Materials (Figures S5 and S6). The measurements were performed for the concentration range from 9.99 × 10^−7^ to 9.09 × 10^−5^ mol/dm^3^.

For each of the polyphenols, the measurements were performed five times, on a newly prepared sensor each time. Standard lines were determined from the average values of peak currents. Standard deviations and error values were calculated. The R^2^ coefficient, sensitivity, precision, accuracy, RSD, and recovery were calculated for the assays performed. The lower limit of detection (LOD) was calculated from the relationship between the slope and the standard deviation of the intercept (LOD = s_b_/a). The calculated values are listed in Table 2. The presented parameters show that, in all cases, the GCE/PEDOT-PSSLi/chit-AuNPs-GA/Laccase sensor worked adequately and was suitable for determining polyphenols. The amperometric method showed the highest sensitivity and allowed for the determination of catechol in higher concentration ranges, from 19 to 90 µM. DPV assays for all three polyphenols allowed for the detection of much lower concentrations. In the case of gallic acid, it was possible to determine it in the range from 2 to 18 µmol/dm^3^; for higher concentrations, the dependence on the concentration was no longer linear. The best results were obtained for the determination of catechol and caffeic acid, for which the linear range was from 2 to 90 µmol/dm^3^, and the sensitivity of these determinations was higher than that for gallic acid. The DPV voltammetry method allowed for the determination of polyphenols in a wider concentration range, with lower LOD values than the amperometric method. Moreover, the amperometric method is troublesome in practical application. The measurements are less reproducible, and the operation of the sensor for a long time, mixed with the solution, often causes much faster wear of the sensor and, thus, the need to repeat the entire procedure anew. Very often, the layer peels off from the substrate. When using DPV as a measurement method, the determination is performed without mixing it with the solution and takes much less time than the amperometric measurement. Sensors in this case are much more durable, which allows for longer use. Cases of detachment of the layer from the substrate occurred sporadically. For these reasons, it was decided to present the results of DPV measurements for all tested polyphenols, and the amperometric method was used only for catechol.

Table 3 shows examples of electrochemical sensors with immobilized laccase. Sensors with different types of detection are presented, both amperometric and voltammetric, as well as sensors with DPV and SWV detection. Compared to these electrodes, the sensors developed by us are characterized by a wide linear range of concentrations. The widest concentration ranges were achieved for the determination of catechol and caffeic acid. For this reason, the developed sensor may be an interesting proposal as a convenient tool for the determination of polyphenols.

**Figure 11:**
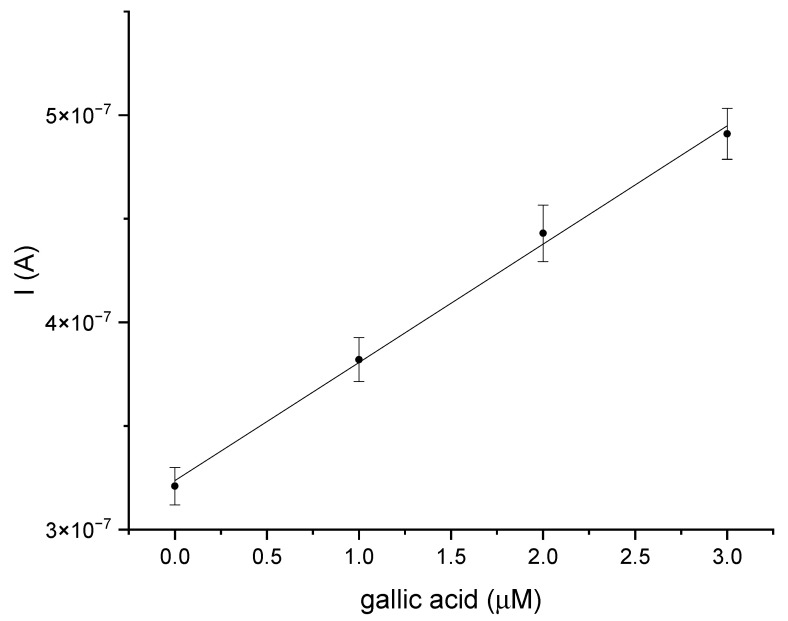
The dependence of the current value on the concentration of gallic acid, obtained using the standard addition method for the GCE/PEDOT-PSSLi/Chit-AuNPs-GA/laccase sensor in white wine samples.

The last stage of the research was an attempt to determine gallic acid in a natural sample, for which white wine was used. The analysis was performed using the GCE/PEDOT-PSSLi/Chit-AuNPs-GA/laccase sensor in phosphate–citrate buffer, pH = 5. This analysis was carried out in order to estimate the accuracy of the presented biosensor. DPV measurements were carried out using the standard addition method. There are DPV curves for this measurement in the Supplementary Materials (Figure S7). Figure 11 shows the dependence of the peak current value on the increasing amount of added gallic acid in the standard addition method for the sensor in a white wine sample. The results for the determination of gallic acid on the GCE/PEDOT-PSSLi/Chit-AuNPs-GA/laccase electrode were as follows. The linear regression equation was expressed as y (A) = 5.71 × 10^−8^ × (µmol/dm^3^) + 3.24 × 10^−7^ (R^2^ = 0.997). The relative standard deviation was 0.96%, and the estimated concentration in the sample was 5.66 µmol/dm^3^. The peak current measurements for each standard addition were performed three times. The standard line was determined from the average values of peak currents, and the results, with a confidence interval for a probability (p) of 95%, were analyzed using linear least-square regression. After the calculations, the content of gallic acid in the determined wine was 4.8 mg/dm^3^. After careful examination of the presented results, it can be concluded that the presented biosensors may be suitable tools for measuring the concentration of gallic acid in real samples, such as white wine. As a reference method in selected natural samples, the spectrophotometric method for determining the concentration of gallic acid, as described by Inoue and Hagerman [27] was used. The idea behind this method is the reaction between rhodamine and the detected polyphenol. The color change in the sample is assessed by measuring the absorbance at l = 520 nm. The content of gallic acid in the determined wine was 4.8 mg/dm^3^. The content of gallic acid in the wine as obtained with the reference method was 4.63 mg/dm^3^. The calculated recovery for the assay performed was 103.67%.

**Table 3:** Examples of different electrochemical sensors with immobilized laccase for the determination of polyphenols.

Phenolic Compound	Sensor	Method	LOD [µM]	Linear Range [µM]	Ref.
Caffeic acid Rosmarinic acid Gallic acid	graphite/ePDA-Lac	amperometry	0.14 0.09 0.29	1–50 1–20 1–150	[28]
Gallic acid	TvL-MWCNTs-SPEd	amperometry	0.6	0.6–99.9	[29]
Catechol	GCE /FYSSns-2-Lac	DPV	1.6	12.5–450	[30]
Caffeic acid Rosmarinic acid Gallic acid	AuSPE/laccase/Nafion	amperometry	2.5 2.4 1.55	3–15 3–15 2–7	[31]
Caffeic acid Rosmarinic acid	Au/Lacc-CS-MWCNT	amperometry	0.151 0.233	0.735–10.5 0.91–112.1	[32]
Catechol	(CNTs–CS)	voltammetry	0.66	1.2–30	[33]
Caffeic acid	Graphite/Lac	amperometry	0.56	1–10	[34]
Mixtures of catechin and caffeic acid	Polyethersulfone/Lac	voltammetry	0.18	12–14	[35]
Catechol	PEI-AuNP-Lac	SWV	0.03	0.36–11	[36]
Caffeic acid Gallic acid	Carbon-Sonogel/Nafion-Lac	amperomtery	0.06 0.41	0.04–2.2 0.01–22	[37]
Catechol Catechol Gallic acid Caffeic acid	GCE/PEDOT-PSSLi/Chit-AuNPs-GA/laccase	amperomtery DPV DPV DPV	9.5 1.7 1.7 1.9	19–90 2–90 2–18 2–90	This work

## 4. Conclusions

The developed sensor is a skillful combination of materials including PEDOT, chitosan, AuNPs, and laccase. PEDOT is a modern conductive polymer ensuring fast charge exchange. Chitosan, as a product of natural origin, is characterized by high biocompatibility, which is of great importance when contact with natural samples is necessary. At the same time, chitosan enables the covalent attachment of the enzyme. On the one hand, AuNPs are able to ensure a sufficiently high conductivity of the chitosan layers; on the other hand, they are also characterized by high biocompatibility. Laccase is an enzyme with a large spectrum of specificity that works as a mediator in the reaction with the analyte. This article described the research leading to the development of the sensor /PEDOT-polystyrene sulfonate)/chitosan-AuNPs-glutaraldehyde/Lacasse. This sensor was designed for the electrochemical determination of polyphenols. The method of manufacturing the sensor was optimized. The effect of the addition of gold nanoparticles on the performance of the sensor, including the possibility of direct oxidation of the immobilized laccase, was described. The influence of AuNPs on the increase in the electrical conductivity of the chitosan layers was presented. A catalytic effect from laccase was demonstrated in the oxidation reaction of polyphenols, using a sensor with an immobilized enzyme. The developed sensor, for the electrochemical determination of catechol using the amperometric method, was used. That sensor was used for the electrochemical determination of polyphenols such as catechol, gallic acid, and caffeic acid with the DPV method. The results of our investigations showed that the sensor can be applied for the determination of these polyphenols. Linear proportional relationships of peak currents to the concentrations of polyphenols were obtained. The determination of gallic acid in wine samples was also conducted, demonstrating the possibility for the practical application of the developed sensor. The sensor had a shelf life of 30 days when stored at 4 °C. It can therefore be concluded that a good analytical tool for the determination of polyphenols has been obtained.

## Figures and Tables

**Table 1:** The roughness parameters for the surfaces of the investigated materials.

	GCE/PEDOT-PSSLi/Chit-GA	GCE/PEDOT-PSSLi/Chit-AuNPs-GA	GCE/PEDOT-PSSLi/Chit-AuNPs-GA /Laccase
Image Z Range [nm]	2.22	1.84	2.89
Image Surface Area [nm^2^]	11,266	11,357	12,010
Image Projected Surface Area [nm^2^]	10,000	10,000	10,000
Surface extension coefficient	1.1266	1.1357	1.2010
Image Surface Area Difference	12.7	13.6	20.1
Image R_q_ [nm]	0.264	0.188	0.447
Image R_a_ [nm]	0.202	0.146	0.361
Image R_max_ [nm]	2.22	1.84	2.89

Rq—Quadratic mean, or root mean square average of profile height deviations from the mean line. Ra—Average, or arithmetic average of profile height deviations from the mean line. R max—The Maximum Roughness Depth is the greatest single roughness depth within the evaluation length.

**Table 2:** Parameters for the determination of polyphenols.

Lp	Polyphenol—Method	R^2^	Sensitivity [A/mol/dm^3^]	CV [%]	Recovery [%]	RSD [%]	LOD [µM]	Linear Range [µM]
1	Catechol—Amperometric	0.996	0.0248	0.082	99.1	15.64	9.5	19–90
2	Catechol—DPV	0.998	0.00508	0.002	100.1	22.83	1.7	2–90
3	Caffeic acid—DPV	0.999	0.0066	0.020	108.7	5.77	1.9	2–90
4	Gallic acid—DPV	0.993	0.0039	0.111	114.7	5.80	1.7	2–18

## Data Availability

All of the involved data are available upon request.

## Supplementary Materials

The following supporting information can be downloaded at https://www.mdpi.com/article/10.3390/ma16145113/s1. Figure S1. (A) SEM image of gold nanoparticles obtained in the synthesis with sodium borohydride. (B) Size distribution histogram of the obtained AuNPs. Figure S2. Voltammetric curves obtained on GCE in a solution of gallic acid C = 0.001 M in phosphate-citrate buffer different pH (v = 200mV/s). Figure S3. Voltammetric curves obtained on GCE in a solution of caffeic acid C = 0.001 M in phosphate-citrate buffer different pH (v = 200mV/s). Figure S4. (A) Amperometric curves for sensor GCE/PEDOT-PSSLi/Chit-AuNPs-GA/laccase in solutions of catechol in phosphate-citrate buffer pH = 5.0. (B) Standard line for catechol. Figure S5. (A) DPV voltammetry curves for catechol solutions in phosphate-citrate buffer pH = 5.0. (B) Standard line for catechol. Figure S6. (A) DPV voltammetry curves for gallic acid solutions in phosphate-citrate buffer pH = 5.0. (B) Standard line for gallic acid. Figure S7. DPV voltammetry curves of gallic acid were obtained using the standard addition method for the GCE/PEDOT-PSSLi/Chit-AuNPs-GA/laccase sensor in white wine samples.
