# Supplementary material for: The Composite Material of (PEDOT-Polystyrene Sulfonate)/Chitosan-AuNPS-Glutaraldehyde/as the Base to a Sensor with Laccase for the Determination of Polyphenols
Source: Materials (Basel). 2023 Jul 20;16(14):5113. doi: 10.3390/ma16145113 (PMC10385068; doi:10.3390/ma16145113)
Supplement: Supplementary file 1 [file materials-16-05113-s001.zip › materials-2455397-supplementary.pdf]

Supporting Information

The Composite Material of (PEDOT-Polystyrene Sulfonate)/Chitosan-AuNPS-Glutaraldehyde/as the Base to a Sensor with Laccase for the Determination of Polyphenols

Paweł Krzyczmonik [1,*], Marta Klisowska [1], Andrzej Leniart [1], Katarzyna Ranoszek-Soliwoda [2], Jakub Surmacki [3], Karolina Beton-Mysur [3] and Beata Brożek-Płuska [3]

[1] Department of Inorganic and Analytical Chemistry, Faculty of Chemistry, University of Lodz, Tamka 12, 91-403 Lodz, Poland

[2] Department of Materials Technology and Chemistry, Faculty of Chemistry, University of Lodz, Pomorska 163 Street, 90-236 Lodz, Poland; katarzyna.ranoszek.soliwoda@chemia.uni.lodz.pl

[3] Laboratory of Laser Molecular Spectroscopy, Faculty of Chemistry, Institute of Applied Radiation Chemistry, Lodz University of Technology, Wroblewskiego 15, 93-590 Lodz, Poland; jakub.surmacki@p.lodz.pl (J.S.)

* Correspondence: pawel.krzyczmonik@chemia.uni.lodz.pl

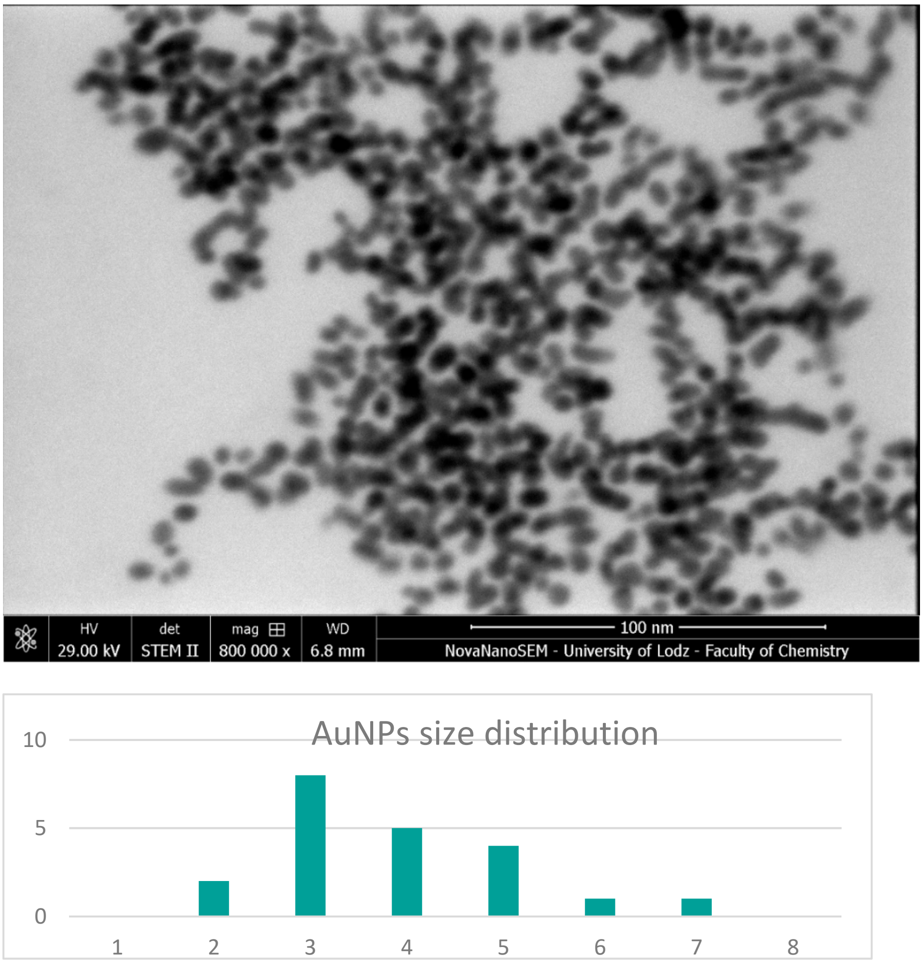

Figure S1. (A) SEM image of gold nanoparticles obtained in the synthesis with sodium borohydride. (B) Size distribution histogram of the obtained AuNPs.

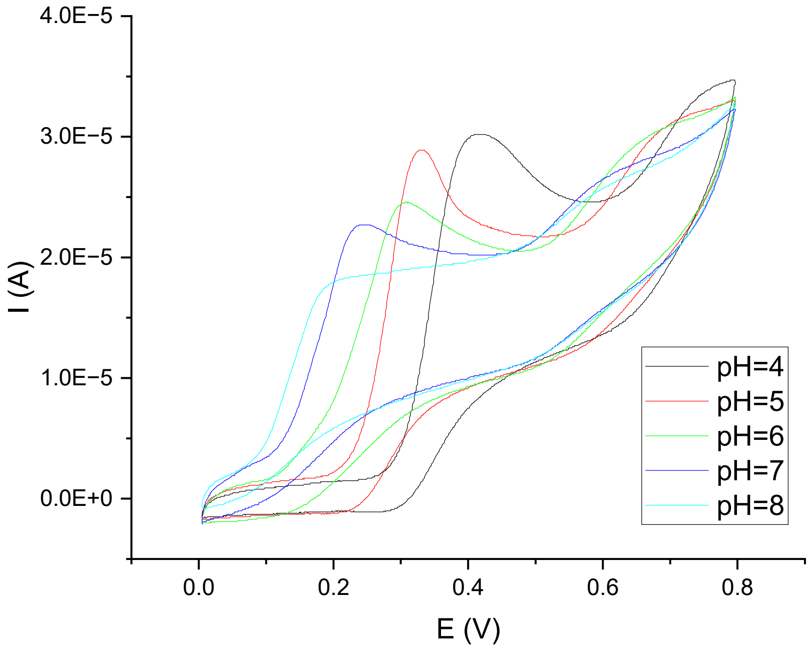

Figure S2. Voltammetric curves obtained on GCE in a solution of gallic acid C = 0.001 M in phosphate-citrate buffer different pH (v=200mV/s). pH=4.0 (black), pH=5.0 (red), pH=6.0 (green), pH=7.0 (blue), pH=8.0 (cyan).

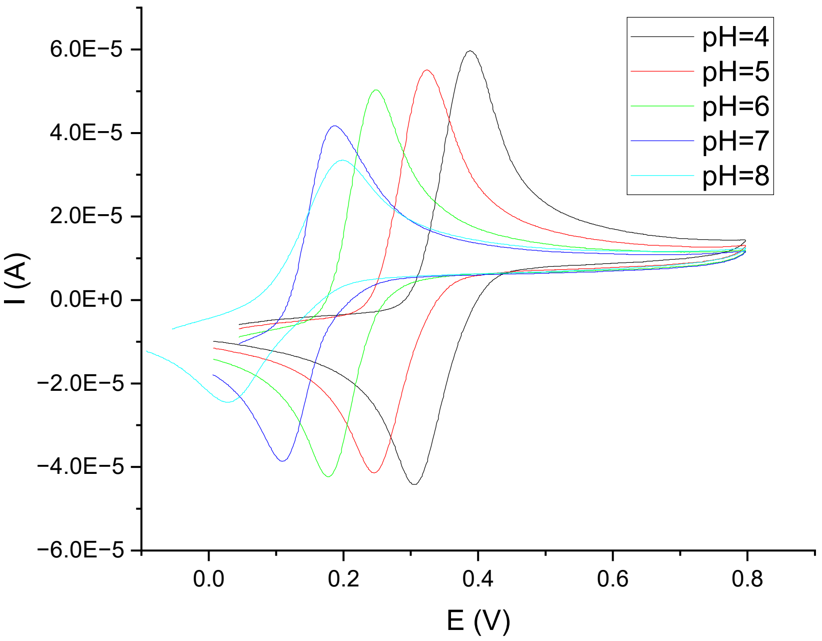

Figure S3. Voltammetric curves obtained on GCE in a solution of caffeic acid C = 0.001 M in phosphate-citrate buffer different pH (v=200mV/s). pH=4.0 (black), pH=5.0 (red), pH=6.0 (green), pH=7.0 (blue), pH=8.0 (cyan).

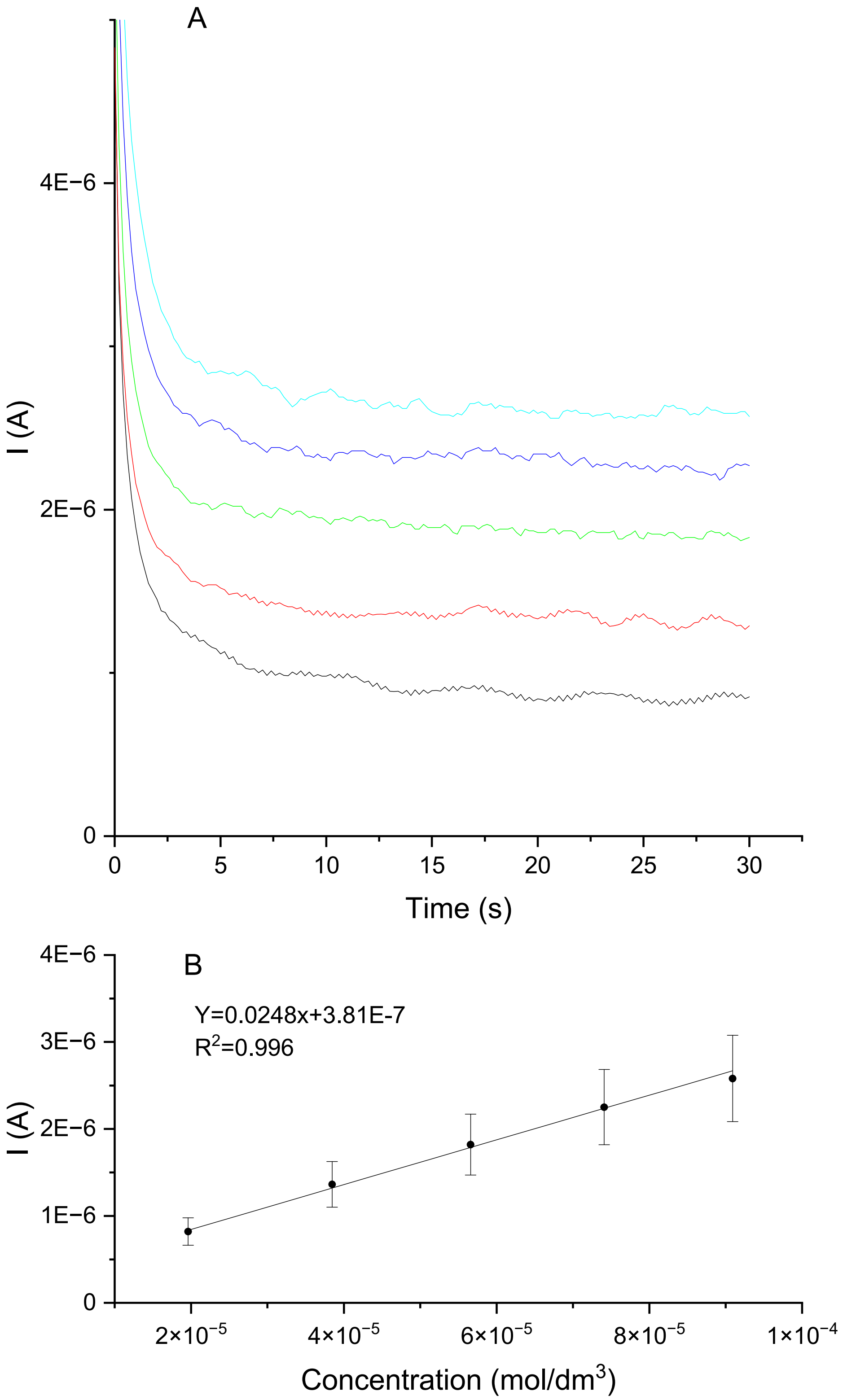

Figure S4. (A) Amperometric curves for sensor GCE/PEDOT-PSSLi/Chit-AuNPs-GA/laccase in solutions of catechol in phosphate-citrate buffer pH = 5.0. (B) Standard line for catechol.

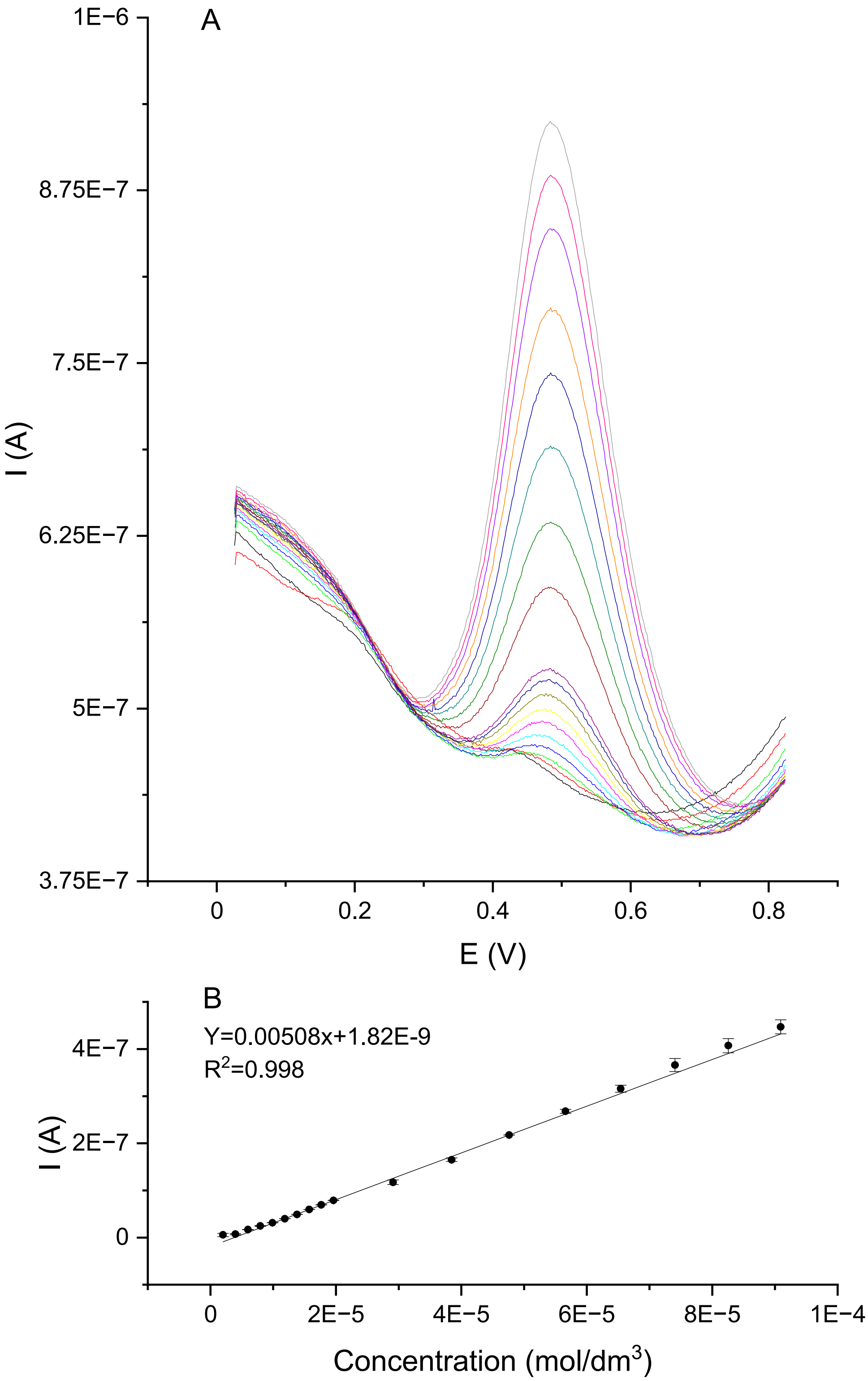

Figure S5. (A) DPV voltammetry curves for catechol solutions in phosphate-citrate buffer pH = 5.0. (B) Standard line for catechol.

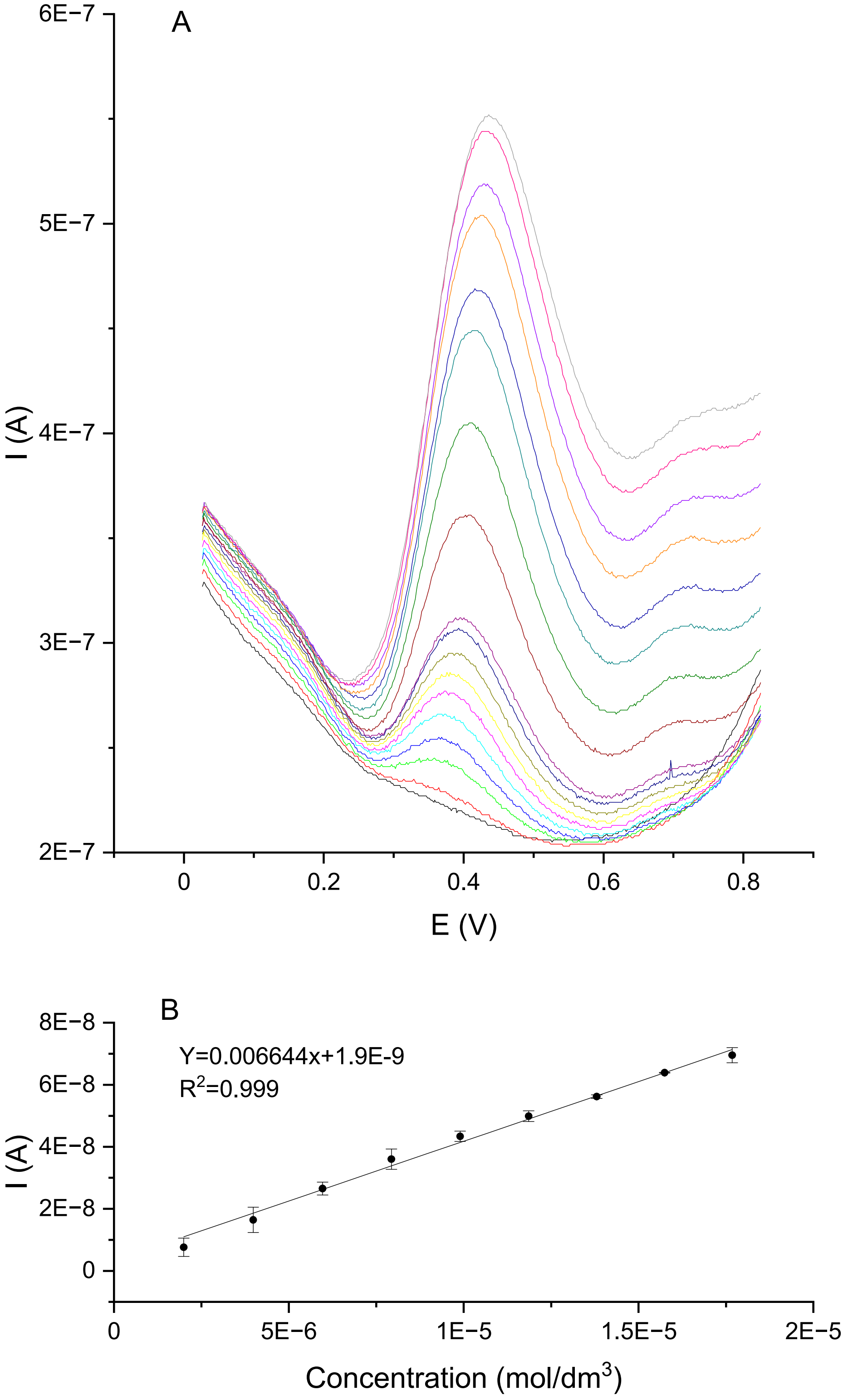

Figure S6. (A) DPV voltammetry curves for gallic acid solutions in phosphate-citrate buffer pH = 5.0. (B) Standard line for gallic acid.

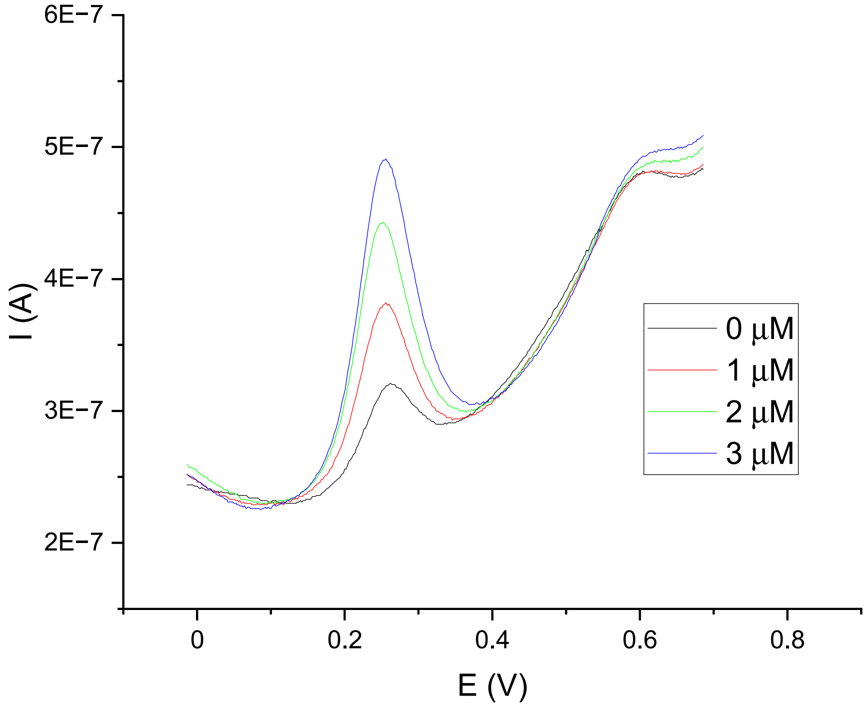

Figure S7. DPV voltammetry curves of gallic acid obtained using the standard addition method for the GCE/PEDOT-PSSLi/Chit-AuNPs-GA/laccase sensor in white wine samples. The standard addition was 1μM.
